# Mobility predicts change in older adults’ health-related quality of life: evidence from a Vancouver falls prevention prospective cohort study

**DOI:** 10.1186/s12955-015-0299-0

**Published:** 2015-07-15

**Authors:** Jennifer C. Davis, Stirling Bryan, John R. Best, Linda C. Li, Chun Liang Hsu, Caitlin Gomez, Kelly A. Vertes, Teresa Liu-Ambrose

**Affiliations:** Centre for Clinical Epidemiology and Evaluation, University of British Columbia and Vancouver Coastal Health Research Institute (VCHRI), 828 West 10th Avenue, Vancouver, BC V6T 2B5 Canada; Department of Physical Therapy, University of British Columbia, 2177 Wesbrook Mall, Vancouver, BC V6T 2B5 Canada; Arthritis Research Centre of Canada, 5591 No. 3 Road, Richmond, BC V6X 2C7 Canada; Aging, Mobility, and Cognitive Neuroscience Lab, University of British Columbia, 2211 Wesbrook Mall, Vancouver, BC V6T 2B5 Canada; Djavad Mowafaghian Centre for Brain Health, University of British Columbia & VCHRI, 2215 Wesbrook Mall, Vancouver, BC V6T 1Z3 Canada; Center for Hip Health and Mobility, University of British Columbia & VCHRI, 828 West 10th Avenue, Vancouver, BC V5Z 1E2 Canada

**Keywords:** Mobility, Quality of life, Falls, Older adults

## Abstract

**Background:**

Older adults with mobility impairments are prone to reduced health related quality of life (HRQoL) is highly associated with mobility impairments. The consequences of falls have detrimental impact on mobility. Hence, ascertaining factors explaining variation among individuals’ quality of life is critical for promoting healthy ageing, particularly among older fallers. Hence, the primary objective of our study was to identify key factors that explain variation in HRQoL among community dwelling older adults at risk of falls.

**Methods:**

We conducted a longitudinal analysis of a 12-month prospective cohort study at the Vancouver Falls Prevention Clinic (*n* = 148 to 286 depending on the analysis). We constructed linear mixed models where assessment month (0, 6, 12) was entered as a within-subjects repeated measure, the intercept was specified as a random effect, and predictors and covariates were entered as between-subjects fixed effects. We also included the predictors by sex and predictor by sex by time interaction terms in order to investigate sex differences in the relations between the predictor variable and the outcome variable, the EQ-5D.

**Results:**

Our primary analysis demonstrated a significant mobility (assessed using the Short Performance Physical Battery and the Timed Up and Go) by time interaction (*p* < 0.05) and mobility by time by sex interaction (*p* < 0.05). The sensitivity analyses demonstrated some heterogeneity of these findings using an imputed and a complete case analysis.

**Conclusions:**

Mobility may be an important predictor of changes in HRQoL over time. As such, mobility is a critical factor to target for future intervention strategies aimed at maintaining or improving HRQoL in late life.

## Background

Poor quality of life is universally acknowledged as an adverse health outcome [1]. A more recent shift is recognizing that poor health related quality of life (HRQoL) may be a critical marker of other adverse health outcomes [1]. It may be that poor HRQoL is an indicator of underlying conditions including pain, disability, depression, polipathology and frailty [1–7]. Several older adult populations (i.e., heart failure, ischaemic heart disease, type 2 diabetes, metatstatic prostate cancer, chronic kidney disease, lung cancer and those awaiting movement to residential care) have demonstrated the prognostic importance of HRQoL of life as independent predictors of death and clinical complications [4, 5, 8–10]. Hence, HRQoL is an important outcome measure in the context of healthy aging.

Impaired mobility is associated with lower HRQoL [11]; however, little research has investigated factors that explain variation in HRQoL over time in this population of fallers at risk for poor HRQoL. Falls are a common geriatric syndrome and are the third leading cause of chonic disability worldwide [12] with approximately 30 % of community-dwelling adults aged 65 and older experiencing one or more falls annually [13]. In particular, of the 30 % of community-dwelling seniors who fall, half fall recurrently and are at significant risk for hospitalization, institutionalization, and even death [14–16]. The consequences of falls have a large and potentially detrimental impact on mobility [17]. Importantly, HRQoL is highly associated with mobility impairments in older adults [18–20]. Specifically, functional abilities such as walking are associated with changes in both physical and mental HRQoL [21]. Given that mobility is a key predictor of HRQoL it is critical to assess factors that explain changes in HRQoL over time in fallers.

To date, much of the insight into factors that explain variation in HRQoL is based on cross-sectional data. As such, the literature is relatively devoid of understanding the determinants of change in HRQoL among older adults among a population at high risk for HRQoL decline—fallers. Hence, our primary objective was to determine what factors were significant predictors of change in HRQoL, as measured by the EQ-5D-3 L, over time (i.e., from baseline to 6 to 12 months) among older men and women presenting to the Vancouver Falls Prevention Clinic. Understanding factors that explain variation in HRQoL over time will help guide future intervention strategies that are aiming to improve HRQoL among older adults at high risk of falls. Women consistently report lower HRQoL compared with men [22]. Hence, our secondary objective was to examine whether or not there was a significant sex by time interaction once key predictors in HRQoL were identified.

## Methods

### Study design

We conducted a longitudinal analysis of a 12-month prospective cohort study at the Vancouver Falls Prevention Clinic (www.fallclinic.com) from June 7, 2010 through October 24, 2013. Participants received a comprehensive assessment at the Vancouver Falls Prevention Clinic at baseline and 12-months. No intervention beyond the comprehensive assessment at the Vancouver Falls Prevention Clinic was received by participants. However, some participants may receive additional followup by the geriatrician or other health care professionals based on recommendations and referrals from the initial assessment.

### Participants

The sample consisted of community dwelling women and men who lived in the lower mainland region of British Columbia were eligible for study entry if they:

- were adults ≥ 70 years of age referred by a medical professional to the Falls Prevention Clinic as a result of seeking medical attention for a non-syncopal fall in the previous 12 months;
- understood, spoke, and read English proficiently;
- had a Physiological Profile Assessment (PPA) [23] score of at least 1.0 SD above age-normative value or Timed Up and Go Test (TUG) [24] performance of greater than 15 s or one additional non-syncopal fall in the previous 12 months (a fall was defined as “Unintentionally coming to the ground or some lower level and other than as a consequence of sustaining a violent blow, loss of consciousness, sudden onset of paralysis as in stroke or an epileptic seizure” [25];
- were expected to live greater than 12 months (based on the geriatricians’ expert opinion);
- were able to walk 3 m with or without an assistive device; and
- were able to provide written informed consent.

We excluded those with a neurodegenerative disease (e.g., Parkinson’s disease) or dementia, patients who recently had a stroke, those with clinically significant peripheral neuropathy or severe musculoskeletal or joint disease, and anyone with a history indicative of carotid sinus sensitivity (i.e., syncopal falls). We highlight that exclusions for this study were based on clinical grounds. The Falls Prevention Clinic is targeting treatment of older adults at risk of impaired mobility and functional decline specifically. Thus individuals with neurodegenerative disease or dementia are referred to alternate clinics.

Ethical approval was obtained from the Vancouver Coastal Health Research Institute and the University of British Columbia’s Clinical Research Ethics Board (H09-02370). All participants provided written informed consent.

#### Vancouver falls prevention clinic measures

A comprehensive set of measurements relating to mobility and cognitive function that were collected at baseline are described below.

### Comorbidity, activities of daily living and depression

Functional comorbidity index (FCI) was calculated to estimate the degree of comorbidity associated with physical functioning [26]. This scale’s score is the total number of comorbidities. We used the 15-item Geriatric Depression Scale (GDS) [27, 28] to indicate the presence of depression; a score of ≥ 5 indicates depression [29].

### Balance and mobility

Mobility and balance were assessed using the Short Physical Performance Battery (SPPB) [30] and the Timed-Up-and-Go Test (TUG) [31]. For the Short Physical Performance Battery, participants were assessed on performances of standing balance, walking, and sit-to-stand performance. Each component is rated out of four points, for a maximum of 12 points; a score < 9/12 predicts subsequent disability [32]. For the TUG, participants rose from a standard chair, walked a distance of three meters, turned, walked back to the chair and sat down [31]. We recorded the time (in seconds) to complete the TUG, based on the average of two separate trials. A TUG performance time of ≥ 13.5 s correctly classified persons as fallers in 90 % of cases [31].

### Physiological falls risk

Physiological falls risk was assessed using the short form of the Physiological Profile Assessment (PPA). The PPA is a valid and reliable [33] measure of falls risk. Based on a participant’s performance in five physiological domains—postural sway, reaction time, strength, proprioception, and vision—the PPA computes a falls risk score (standardized score) that has a 75 % predictive accuracy for falls in older people [34, 35]. A PPA Z-score of ≥ 0.60 indicates high physiological falls risk [36].

### Global cognitive function

We assessed global cognition using the Mini Mental State Examination (MMSE) and the Montreal Cognitive Assessment (MoCA). The MMSE is a widely used and well-known questionnaire used to screen for cognitive impairment (i.e., MMSE <24) [37]. It is scored on a 30-point scale with a median score of 28 for healthy community dwelling octogenarians with more than 12 years of education [37]. The MMSE may underestimate cognitive impairment for frontal system disorders because it has no items specifically addressing executive function [37].

The Montreal Cognitive Assessment (MoCA), a brief screening tool for MCI [38] with high sensitivity and specificity, was used to categorise participants as with, or without, possible MCI. It is more sensitive than the MMSE in detecting mild cognitive impairment [38]. It is a 30-point test covering eight cognitive domains: 1) attention and concentration; 2) executive functions; 3) memory; 4) language; 5) visuo-constructional skills; 6) conceptual thinking; 7) calculations; and 8) orientation. Scores below 26 are considered to be indicative of possible MCI. A bonus point is given to individuals with less than 12 years of education.

#### Primary outcome measure

The primary outcome variable of interest is the EQ-5D-3L. Patients completed the EQ-5D-3L using paper versions that were given to them upon presentation to the Falls Prevention Clinic. Telephone interviews were used to complete the EQ-5D-3L at 6 and 12 months. No cards were used to aid interpretation.

### EQ-5D-3L

We assessed HRQoL using the EQ-5D three level version (EQ-5D-3L). The EQ-5D-3Lis a preference based utility instrument that captures 243 health states [39] to ascertain an individual’s HRQoL according to five domains: mobility, self-care, usual activities, pain and, anxiety or depression. Each domain has three possible response options indicating no problems, some problems or severe problems. The EQ-5D-3 L health state utility values (HSUVs) at each time point are bounded from −0.54 to 1.00 where a score of less than zero is indicative of a health state worse than death. Individuals’ preferences for the scoring of the EQ-5D-3L were estimated using the time trade off technique on a random sample of adults taken from the population living in the York (UK) region (*N* = 3000) [40]. Thus, the EQ-5D reflects societal norms of individuals’ preferences for a distinct set of health states. The EQ-5D is the most widely used generic instrument that uses a utility-based scoring approach, yielding a single summary score (i.e., health-state utility value) on a common scale to facilitate comparison across different health conditions and patient populations [39]. The HSUV is anchored at zero—a health state equivalent to death, and 1.0—a state of “full health.” Health-state utility values less than zero are defined as health states worse than death. Health-state utility values are an essential outcome for economic evaluations.

### Handling of missing data

Missing data were handled in three ways. First, using the restricted maximum likelihood estimator, all individuals with baseline data for the variables in the model (i.e., available case set) were included (ML analysis). Specifically, an available case set only includes data where results are known, using as a denominator the total number of people who had data recorded a particular variable of interest, models were restricted to those individuals with Escore data at baseline and 12-month follow-up (i.e., complete case analysis). Of note, a complete case analysis deletes all participant IDs with incomplete data (in the variables involved) from the analysis. Third, multiple imputation using the ICE (Imputation by Chained Equations) procedure in STATA 10.0 was using to create five complete data sets (MI analysis). We followed recommendations by Oostenbrink [41, 42] and Briggs [43, 44] for multiple imputation of missing effectiveness data. We imputed missing EQ-5D values at each time point (i.e., 6 and 12 months). For each missing value, we generated five possible values using multiple linear regression. Covariates included age, FCI, TUG, PPA and baseline EQ-5D utility score, and the weight and value of the missing variable in the preceding period. The final imputed value was the mean value from the five data sets created.

### Statistical analyses

We report the available case set as our base case analysis. Data were initially examined using visual analysis of histograms and computation of skew and kurtosis. The TUG and SPPB variables departed from normality (skew > |1|) and underwent log_10_ transformation. The outcome variable, EQ-5D-3 L HSUVs, also showed skew at each time point (ranging between −1.5 and −1.4); therefore, analyses were conducted on the transformed EQ-5D-3 L HSUVs.

For the main analyses, linear mixed models were constructed using the SPSS 22.0 MIXED procedure (IBM Corporation, 2013). Assessment month (0, 6, 12) was entered as a within-subjects repeated measure, the intercept was specified as a random effect, and predictors (i.e., SPPB or TUG, depending on the model) and covariates (i.e., sex, age, and their interactions with time (i.e., sex*time, age*time)) were entered as between-subjects fixed effects. A first-order auto-regressive covariance matrix provided superior model fit compared to an unstructured covariance matrix (based on the Bayesian Information Criterion) and allowed for model convergence across the models. Denominator degrees of freedom were calculated from the Satterthwaite approximation [45].

A separate linear mixed model was constructed for each predictor variable examined. In addition to the specific predictor and its interaction with time, models include participant age and sex and their interactions with time. We also included the predictor X sex and predictor X sex X time interaction terms in order to investigate sex differences in the relations between the predictor variable and the outcome variable, EQ-5D-3 L HSUVs. If not statistically significant, these terms were dropped. Additionally, in the examination of SPPB and TUG as key mobility related predictors, the use of armrest was included as a covariate, along with its interaction with time. The use of armrest did not interact with the main variables of interest the model, and therefore these interaction terms were excluded. In the text, we report the unstandardized beta estimate (*B*), its 95 % confidence interval, and its significance value. Given a significant interaction with sex, we stratified the data and ran the models separately for males and females. To visualize significant interaction effects, we used model-based estimated marginal means at low (−1 *SD*), average (0 *SD*), and high (+1 *SD*) levels of the predictor [46]. When a higher-order interaction was significant (e.g., 3-way interaction), we do not report significant lower-order interactions (2-way interactions) or main effects.

## Results

Two-hundred and forty three (for the SPPB) or 244 (for the TUG) participants are included in the Maximum Likelihood Models using the available case analysis. Our sensitivity analyses include the complete case analysis (*n* = 148) and the imputed case analysis (*n* = 286). Further, we also conducted all of the above analyses using the log_10_ transformed EQ-5D-3 L HSUVs.

### Participants

Table 1 reports descriptive statistics of the complete case analysis at baseline for our variables of interest for this cohort. At baseline, this cohort of community-dwelling senior women has a mean (SD) EQ-5D-3 L HSUV of 0.78 (0.22), a mean SPPB of 7.3 (2.5) and a mean TUG of 19.7 (10.5). On average, participants had at least two existing co-morbidities and were 82 ± 7 years of age. Participants were classified as having high falls risk with a mean PPA score of 1.6 ± 1.0. Further, the mean MMSE score was 26 ± 3 and the mean MoCA score was 22 ± 4. A cut-off of 26 or lower on the MoCA is used to classify individuals with mild cognitive impairment.

**Table 1:** Baseline characteristics of the Vancouver falls prevention cohort (available case analysis)

Variables at baseline	Mean (SD) or number (%) or median (IQR)
Age (years) (*n* = 315)	82.5 (6.5)
Sex (Male/Female) (*n* = 308)	112 (36.4)/196 (63.4)
Living status (*n* = 253)	
Lives alone	100 (39.5)
Lives with others	122 (48.2)
Assisted living	31 (12.3)
Education (*n* = 299)	
< Grade 9	33 (11.0)
Grades 9–13, no diploma	59 (19.7)
High school with diploma	58 (19.4)
Trades school	23 (7.7)
Some university	36 (12.0)
University	90 (30.1)
FCI (*n* = 320)	2.5 (1.9)
GDS (*n* = 315)	3.1 (2.6)
EQ-5D (*n* = 245)	0.778 (0.217) or 0.8 (0.27)
SPPB (*n* = 303)	7.3 (2.5)
TUG (*n* = 296)	19.7 (10.5)
PPA (*n* = 311)	1.7 (1.1)
MMSE (*n* = 315)	26.4 (3.2)
MoCA (*n* = 303)	22.1 (4.6)

### Available case analysis (non-transformed EQ-5D)

The base case analysis is presented in Table 2.

**Table 2:** The maximum likelihood model for the available case analyses for the SPPB and TUG

	Maximum likelihood	
Predictor	Non-transformed	Log-transformed
SPPB, *N* = 243	*B* (*p* value)	*B* (*p* value)
SPPB	.04 (<.001)**	.12 (<.001)**
SPPB*time	−.03 (.045)*	−.08 (.041)*
SPPB*sex	−.01 (.239)	−.04 (.328)
SPPB*sex*time	.04 (.036)*	.10 (.077)
TUG, *N* = 244		
TUG	−.41 (<.001)**	−1.29 (<.001)**
TUG*time	.33 (.032)*	.94 (.053)
TUG*sex	.19 (.329)	.50 (.414)
TUG*sex*time	−.57 (.040)*	−1.48 (.084)

*p < 0.05

**p < 0.01

#### Short performance physical battery

**Fig. 1:**
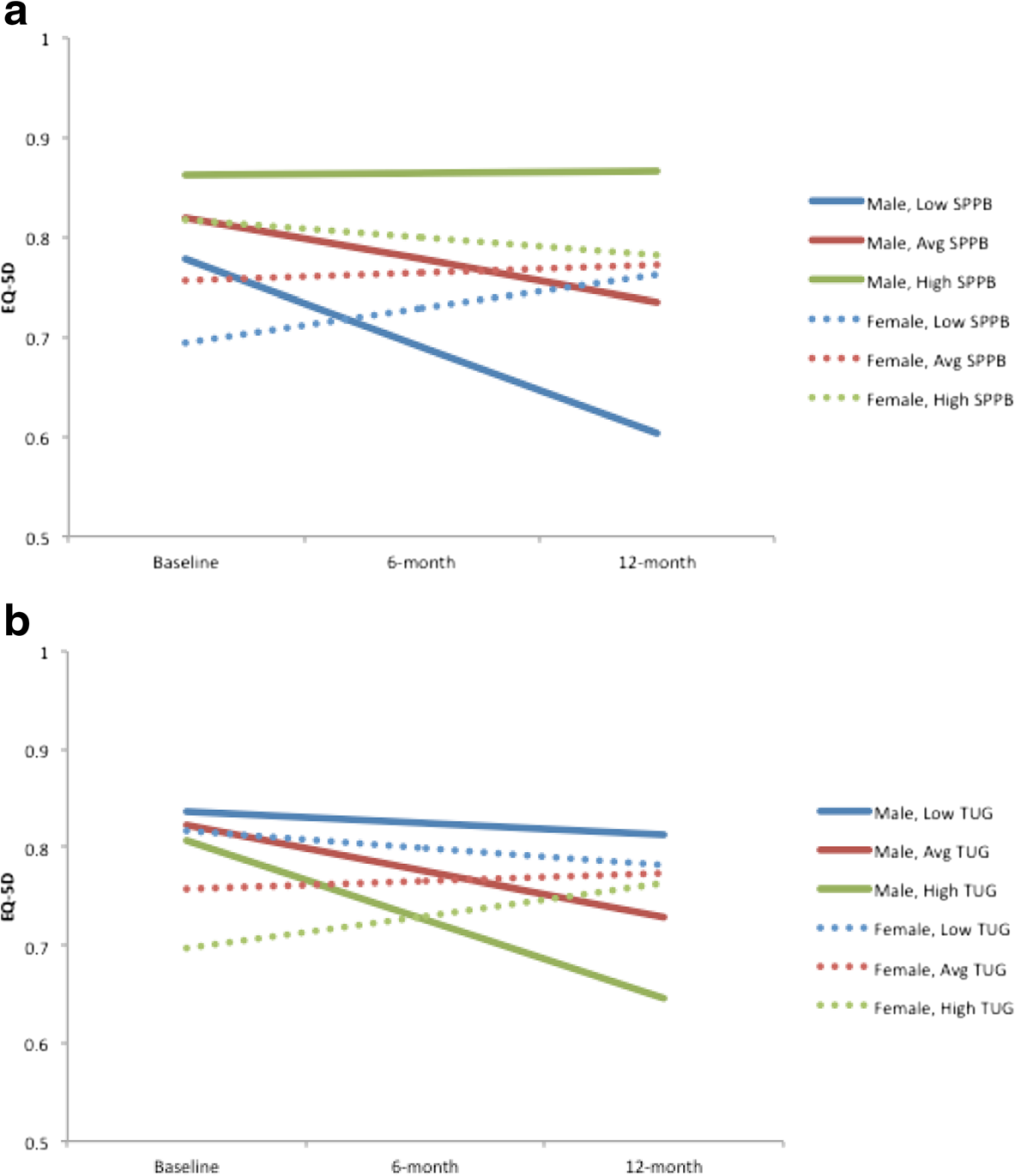
**a** SPPB by time interaction among men and women over 12 months. **b** TUG by time interaction among men and women over 12-months

**Fig. 2:**
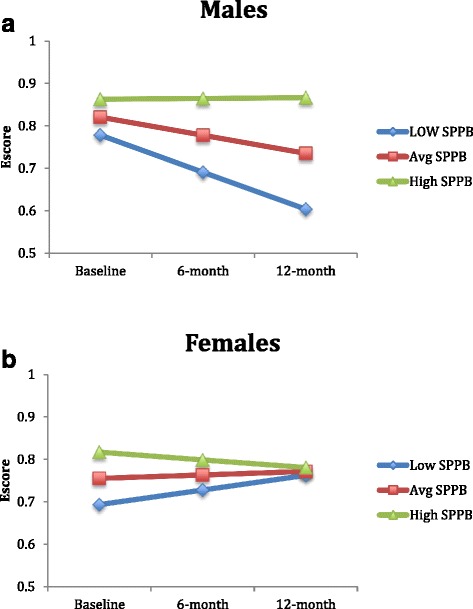
**a** Model-based estimated marginal means for low (−1 SD), average and high (+1 SD) SPPB scores for males. **b** Model-based estimated marginal means for low (−1 SD), average and high (+1 SD) SPPB scores for females

The Maximum Likelihood Model for the available case analysis (*n* = 243) demonstrated that baseline SPPB was associated with baseline EQ-5D HSUVs. Further, a significant SPPB by time interaction (*p* < 0.05) and SPPB by time by sex interaction (*p* < 0.05) were also observed (Fig. 1a and b). When the analyses were run separately for males and females using the complete case set, we found that for males (*n* = 51), baseline SPPB was not associated with baseline EQ-5D HSUVs (*B* = .02, *p* = .295) but there was a trend for SPPB to predict change in EQ-5D HSUVs over time (*B* = .04, *p* = .081). Alternatively, for females (*n* = 97), baseline SPPB was associated with baseline EQ-5D HSUVs (*B* = .03, *p* = .034) but did not predict change in EQ-5D HSUVs over time (*B* =−.02, *p* = .127). These effects among men and women are graphed in Fig. 2a and b.

#### Timed up and go

**Fig. 3:**
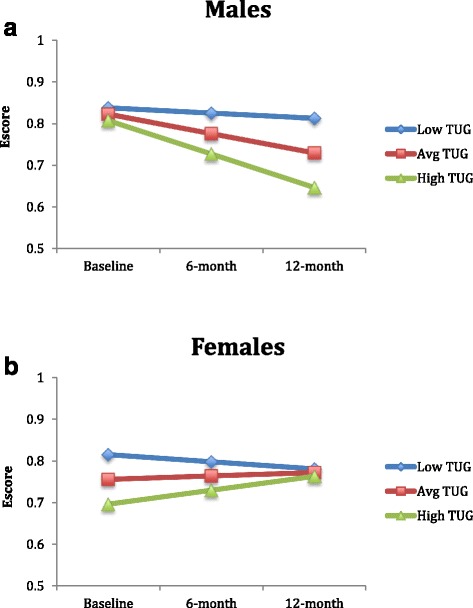
**a** Model-based estimated marginal means for low (−1 SD), average and high (+1 SD) TUG scores for males. **b** Model-based estimated marginal means for low (−1 SD), average and high (+1 SD) TUG scores for females

The Maximum Likelihood Model for the available case analysis (*n* = 244) demonstrated that baseline TUG was associated with baseline EQ-5D HSUVs. Further, a significant TUG by time interaction (*p* < 0.05) and TUG by time by sex interaction (*p* < 0.05) were also observed for the non-transformed EQ-5D data. When the analyses were run separately for males and females using the complete case set, we found that for males (*n* = 57), baseline TUG was not associated with baseline EQ-5D HSUVs (*B* = -0.10, *p* = .671) nor change in EQ-5D HSUVs over time (*p* = 0.139). For females (*n* = 91), baseline TUG was associated with baseline EQ-5D HSUVs (*B* = -0.33, *p* = .015) and there was a trend for TUG predicting change in EQ-5D HSUVs over time (*B* = 0.28, *p* = .073). These effects among men and women are graphed in Fig. 3a and b.

### Sensitivity analysis

All sensitivity analyses were conducted on the transformed and non-transformed EQ-5D data are presented in Table 3.

**Table 3:** Mixed linear models for the multiply imputed and complete case sets for the SPPB and TUG

	Multiple imputation		Complete case	
	*N* = 286		*N* = 148	
Predictor	Non-transformed	Log-transformed	Non-transformed	Log-transformed
SPPB	*B* (*p* value)	*B* (*p* value)	*B* (*p* value)	*B* (*p* value)
SPPB	.03 (<.001)**	.10 (<.001)**	.02 (.052)	.08 (.042)*
SPPB*time	−.01 (.076)	−.03 (.087)	−.01 (.388)	−.04 (.312)
SPPB*sex	−.01 (.348)	−.03 (.462)	−.002 (.926)	.001 (.979)
SPPB*sex*time	.01 (.304)	.02 (.422)	.04 (.036)*	.12 (.067)
TUG	*N* = 290		*N* = 148	
TUG	−.36 (<.001)**	−1.13 (.001)**	−.27 (.042)*	−.89 (.039)*
TUG*time	.11 (.075)	.31 (.111)	.23 (.158)	.64 (.209)
TUG*sex	.07 (.716)	.11 (.852)	.11 (.652)	.20 (.792)
TUG*sex*time	−.16 (.169)	−.41 (.274)	−.70 (.015)*	−1.94 (.032)*

*p < 0.05

**p < 0.01

### Complete case analysis

#### Short performance physical battery

The Mixed Linear Model for the complete case analysis (*n* = 148) demonstrated that baseline SPPB was associated with baseline EQ-5D HSUVs for the log-transformed EQ-5D data only. Further, a significant SPPB by time interaction (*p* < 0.05) and SPPB by time by sex interaction (*p* < 0.05) were not observed for the transformed or non-transformed data. For the EQ-5D data (non-transformed), a significant SPPB by sex by time interaction was observed (*p* < 0.05).

#### Timed up and go

The Mixed Linear Model for the complete case analysis (*n* = 148) demonstrated that baseline TUG was associated with baseline EQ-5D HSUVs. Further, a significant TUG by time by sex interaction (*p* < 0.05) was also observed for the transformed and non-transformed EQ-5D data.

### Imputed case analysis

#### Short performance physical battery

The Mixed Linear Model for the imputed case analysis (*n* = 286) demonstrated that baseline SPPB was associated with baseline EQ-5D HSUVs for both the transformed and non-transformed data. No significant SPPB by time interaction (*p* > 0.05) and SPPB by time by sex interaction (*p* > 0.05) were observed for the transformed and non-transformed EQ-5D data.

#### Timed up and go

The Mixed Linear Model for the imputed case analysis (*n* = 286) demonstrated that the baseline TUG was associated with baseline EQ-5D HSUVs. No significant TUG by time interaction (*p* > 0.05) and TUG by time by sex interaction (*p* > 0.05) were observed for the transformed and non-transformed EQ-5D data.

## Discussion

HRQoL is an essential component that contributed to healthy ageing [47]. Given the demonstrated association between mobility and HRQoL [18–20], it is critical to understand key measures that explain variations in HRQoL among high risk groups such as older fallers. In this study, we found that two valid and reliable measures of mobility—the SPPB and the TUG—predicted HRQoL over time and this relationship was dependent on sex in a population of older fallers. As such, mobility may be a critical measure to consider to maintain or improve HRQoL among older fallers and promote healthy ageing.

This study extends our previous cross-sectional findings that the SPPB, a valid and reliable measure of balance and mobility, explained a large and significant amount of variation in HRQoL at baseline [48]. We now demonstrate, for males, there was a significant SPPB by time interaction indicating that the change in SPPB over time explains significant variation in HRQoL. Conversely, this was not observed for females. Specifically, the average trajectories for males of low, medium and high SPPB scores all demonstrated a trend of decline over the 12 month period with the high SPPB group experiences the slowest rate of decline. For females, there appeared to be a regression to the mean effect (i.e., regardless of baseline function, over time, all females demonstrated a trend toward the average), with the high SPPB group declining over time and the low and average SPPB groups improving over time. Examining the underlying reasons for these differences in balance and mobility and their change over time between males and females is essential to appropriate target intervention strategies. One hypothesis is that individuals with low and average SPPB scores may be more compliant with the recommendations received at the Falls Prevention Clinic.

The intricacies of sex specific relationships between mobility and HRQoL over time are largely understudied. One previous cross-sectional study demonstrated that a low SPPB score was significantly associated with the lowest quartile of EQ-5D index score for men and lowest and second lowest quartiles for women [49]. Gait speed was significantly associated with the EQ-5D index for participants of both sexes, however, standup time was associated with the EQ-5D for men only [49]. As such, cross-sectional data have previously demonstrated sex effects in the relationship of balance and mobility with HRQoL [47]. Despite these associations, there remains a gap in the understanding of longitudinal changes in mobility and the unique contribution to HRQoL among men and women.

This study provides a critical first step for future longitudinal studies and intervention studies to explore the temporal relationships of mobility and HRQoL among men and women and to consider the targeting of future intervention strategies aimed at improving or maintaining HRQoL differently among men and women. Specifically, we need to better understand why the observed trajectories among men and women demonstrate different temporal trends and how this impacts prevention and treatment strategies delivered by clinicians. For example, compliance to recommendations (a behavioural pattern) between men and women may be an explanatory factor. If so, this would highlight that clinicians need to tailor treatment and management for men and women differently.

We note the following limitations of our study. Although this was not an intervention study, it could be possible that the management of balance and mobility may confound the outcomes. The presence of missing data could influence the interpretation of the results. As such, we conducted sensitivity analyses with the multiple imputed case set and the complete case set. Further, the EQ-5D data were significantly skewed. Although are sample size was large enough that the analyses should be robust to departure from normality, in our base case and sensitivity analyses, we report the result of the transformed and non-transformed data.

## Conclusions

This study confirms the critical role that mobility plays in HRQoL at baseline among older fallers. Further it highlights key differences in this relationship between men and women over time. Specifically, men demonstrate decline over time regardless of mobility status; whereas women in the highest tertile of mobility only demonstrate a declining trend in HRQoL over time. One potential explanation that needs investigation is that women may be more compliant with recommendations received at the Falls Prevention Clinic. The important message at a clinical level is that men and women’s treatment and prevention strategies need to tailored treatment and prevention strategies.
